# Social Predation by a Nudibranch Mollusc

**DOI:** 10.1093/iob/obaf017

**Published:** 2025-04-28

**Authors:** K Otter, S Gamidova, P S Katz

**Affiliations:** Neuroscience and Behavior Graduate Program, University of Massachusetts Amherst, Amherst MA 01002, USA; Department of Biology, University of Massachusetts Amherst, Amherst MA 01002, USA; Neuroscience and Behavior Graduate Program, University of Massachusetts Amherst, Amherst MA 01002, USA; Department of Biology, University of Massachusetts Amherst, Amherst MA 01002, USA

## Abstract

**Synopsis:**

Social predation is a common strategy used by predators to subdue and consume prey. Animals that use this strategy have diverse methods of finding each other, organizing behaviors, and capturing prey. There is wide variation in the extent to which these behaviors are coordinated and in the stability of individual roles. This study characterizes social predation by the nudibranch mollusc, *Berghia stephanieae*, which is a specialist predator that eats only the sea anemone, *Exaiptasia diaphana*. A combination of experimental and modeling approaches established that *Berghia* consistently preys upon *E. diaphana* in groups, even when resources are abundant. However, this preference for social foraging does not appear to be a fixed personality trait, as individuals did not exhibit stable roles such as leader or follower. Instead, the population exhibited fission–fusion dynamics with temporary roles during predation. The extent of this social feeding was not altered by length of food deprivation, suggesting that animals are not shifting strategies based on hunger state. Furthermore, classic gastropod cues—such as slime trails, attraction to injured anemones, or preference for conspecifics feeding—did not facilitate group formation. Thus, *Berghia* provides an example of a specialist predator of dangerous prey that loosely organizes social feeding, independent of hunger state and fixed individual roles, while the mechanism of aggregation remains unknown.

**Significance Statement:**

Social predation is an adaptive strategy that enables predators to subdue dangerous prey while minimizing injury. Many nudibranchs specialize to predate upon cnidarians, which pose unique challenges due to their potent defenses. Although nudibranchs are often characterized as solitary hunters, our study reveals that *Berghia stephanieae* exhibits social predation behaviors, forming temporary, fluid groups to feed on sea anemones. These groups lack stable social structures, with individuals adopting temporary roles such as joining or initiating feeding. Interestingly, we found no evidence that aggregation is driven by simple cues such as slime trails, conspecific activity, or prey injury, suggesting that group formation may depend on more complex or context-specific mechanisms. This work highlights the need for further research into the ecological and sensory factors underlying social predation in nudibranchs and other marine predators.

## Introduction

Social feeding behaviors have been extensively studied across taxa, from simple multicellular organisms such as *Trichoplax adhaerens* to complex animals such as cephalopods and wolves (Krause and Ruxton 2002; MacNulty et al. 2014; Fortunato and Aktipis 2019; Burford and Robison 2020). Feeding in groups can be costly, leading to increased competition for food and the risk of attracting predators (Sutton et al. 2015; Balaban-Feld et al. 2019). However, hunting and feeding in groups often provides key advantages, such as increased efficiency in locating and subduing prey, improved vigilance against predators, and reduced individual handling times during dangerous interactions with prey (Brown and Richardson 1988; Barta et al. 2004; MacNulty et al. 2014). For example, lionfish achieve higher hunting success rates in groups (Lönnstedt et al. 2014; Sarhan and Bshary 2023) and electric eels herd prey for collective electrical strikes (Bastos et al. 2021). A key benefit of social predation is the capacity to subdue and kill larger, more dangerous prey (Brown and Richardson 1988; Mukherjee and Heithaus 2013; MacNulty et al. 2014). Social predation strategies, which range from highly choreographed attacks to loose aggregations, represent diverse adaptations to these trade-offs (Lang and Farine 2017).

Feeding in groups covers a broad continuum, from organized hunts in which individuals have defined roles to loose aggregations attracted to the same resources. Complex social predation strategies, which include choreographed attack patterns, are used by animals that live socially (Berghänel et al. 2022) as well as by animals that are generally solitary (Lührs and Dammhahn 2010; Twining and Mills 2021). Thus, social living is not directly tied to social predation. In contrast, simpler strategies involve aggregation without coordinated behavior, as seen in brown bears feeding at salmon runs (Deacy et al. 2016) and predatory nematode-hunting mites that aggregate around injured prey (Aguilar-Marcelino et al. 2014). This continuum of strategies underscores the importance of studying a wide range of taxa to identify both general principles of group hunting and the unique ecological pressures that drive novel adaptations for coordinated predation. For the purposes of this paper, social predation is defined as encompassing behaviors where predators “find, capture and consume animals with others” (Lang and Farine 2017).

Social predation has not been documented in the Nudipleura (Nudibranchia and the sister taxon Pleurobranchomorpha). Most nudipleurans are solitary predators, and many species exhibit aggressive or even cannibalistic behaviors when encountering conspecifics, such as *Pleurobranchaea californica, Gymnodoris* spp., and *Hermissenda crassicornis* (Megina et al. 2007; Gillette and Brown 2015; Knutson and Gosliner 2022). Interspecific aggression and cannibalism are not only observed in nudipleuran generalist predators, but in some stenophagous nudipleurans including *Chromodoris annae*, a specialist of *Petrosaspongia* sp. sponges, *Glaucus marginatus*, a specialist on colonial hydrozoan cnidarians, and *Godiva quadricolor*, a hydroid specialist (Willan 2010; Huang et al. 2017; Macali et al. 2023). While some species aggregate temporarily for mating (e.g., *Palio dubia* and *Baeolidia moebii*) (Hamel et al. 2008; Kytinou et al. 2022), and others have been observed feeding in groups, these instances typically involve large, sessile, colonial prey rather than active, defended organisms. For example, the aeolid *Phyllodesmium poindimiei* feeds in groups on soft corals, though these corals lack active antipredator behaviors and rely solely on chemical defenses (Wagner et al. 2009). Similarly, *Duvaucelia plebeia* has been documented feeding in groups on large soft corals, though these encounters are marked by conspecific aggression, suggesting resource limitation rather than cooperative feeding (Allmon and Sebens 1988). Other species, such as *Doris verrucosa*, congregate to feed on large sponges (López-Acosta et al. 2023). The rare observation of high densities of *Tritonia hamnerorum* in the Florida Keys only documented in 1992 (Cronin et al. 1995) further highlights how unusual group feeding (GF) is in this clade.

Aeolid nudibranchs often specialize in feeding on hexacorallians, especially anemones (Goodheart et al. 2022). Anemones represent a prey type with more active defensive behaviors than many sessile corals, hydroids, and bryozoans (Edmunds et al. 1976). Some anemones have even been observed capturing and consuming potential nudibranch predators (Lam et al. 2017; Mehrotra et al. 2019; Hayes and Schultz 2022). *Aeolidia papillosa*, for example, primarily consumes sea anemones, including the aggregating anemone *Anthopleura elegantissima*, yet there are no records of multiple individuals feeding on the same anemone (Harris and Howe 1979). This species is known to consume up to 100% of its body weight daily, making GF potentially disadvantageous due to competition for nutrients (Seavy and Muller-Parker 2002). Interestingly, *A. papillosa* generally avoids acontiate anemones, suggesting that acontia—specialized defensive structures that can be quickly extruded from the pedal disc—serve as a particularly potent deterrent (Hall et al. 1984).

*Berghia stephanieae* is a monophagous specialist predator feeding on one species of sea anemone, *Exaiptasia diaphana.* Anemones are both highly defended with acontia and actively capable of injuring or killing their predators (Carroll and Kempf 1990; Monteiro et al. 2020; Goodheart et al. 2022). Given the dangers associated with feeding on *E. diaphana, Berghia* may exhibit social predation as a strategy to reduce the risk of severe injury during predation. This study was inspired by observations of aggregations during routine feedings in the laboratory and in cases where there was an abundance of prey, such as introducing a group of *Berghia* to clear a 10-gallon tank of anemones. Unlike most nudibranchs, *Berghia* can be housed in groups without cannibalism. These observations indicate that *Berghia* may be a more social species of nudibranch and their specialization on dangerous prey provided an ideal opportunity to investigate whether *Berghia* engages in social predation.

Here, we addressed whether *Berghia* feeds socially, aggregating at anemones in a nonrandom manner, and sought to determine the mechanisms underlying this aggregation behavior. In this study, we first experimentally established that *Berghia* feeds socially even with the opportunity to feed individually. Our findings provide the first experimental evidence of social predation in a nudibranch. Building on this finding, we investigated potential mechanisms underlying aggregation. To aggregate, individuals must be using cues from other slugs and/or from the prey. For example, they might be following slime trails left by other slugs or be attracted to cues emitted from conspecifics feeding on an anemone. The latter could indicate an active predation event, which would reduce the costs of searching and injury. Alternatively, they might be attracted to chemicals released from injured anemones, which might indicate vulnerability of the prey.

We also examined whether hunger state influences the likelihood of social feeding. We predicted that intermediate levels of food deprivation would promote GF, reflecting a trade-off between resource acquisition and safety, where intermediately food-deprived animals balance the need to feed with the risk of injury. We predicted that more food-deprived slugs would display less aggregation despite the increased risk of injury in order to maximize energy intake. Finally, we tested whether individual slugs consistently prefer social or solitary feeding, hypothesizing that preferences might vary among individuals, potentially reflecting personality traits or ecological strategies.

## Methods

### Animal care

Our laboratory has established and maintains a colony of *B. stephanieae* derived from individuals purchased from Salty Underground (Crestwood, MO, USA) and Reeftown (Boynton Beach, FL, USA). Prior to use in this study, *Berghia* were communally housed in groups of 5–15 individuals in 1-gallon acrylic aquariums filled with artificial seawater (ASW; Instant Ocean, Blacksburg, VA, USA), made with a specific gravity of 1.020–1.022 and pH of 8.0–8.5 with a 12:12 light–dark cycle at 22–26°C. *Exaiptasia diaphana* (Carolina Biological Supply Co., Burlington, NC, USA) were housed in glass aquariums containing ASW. Unless otherwise noted, the *Berghia* were fed twice a week by placing two *E. diaphana* individuals in their home tank, in accordance with the standard feeding regime used by our laboratory (Quinlan and Katz 2023). The *E. diaphana* used in this experiment had an average oral disc diameter of 0.4 cm (median = 0.39 cm, SD = ±0.14 cm) and the *Berghia* used in this study were reproductive adults (0.5–1 cm in length). The size difference between the slug length and anemone diameter was similar across experiments, with larger slugs size matched with larger anemones. Anemones selected were all a size that could be entirely consumed by an individual adult slug over the course of 24 h. Each animal was used only once, and the only retested individuals were used for the consistency of social preferences assay.

### Group feeding assay

To test whether *Berghia* feed on *E. diaphana* in groups even if they have the option to feed alone, groups of *Berghia* were provided with a set of equally distant anemones with a 1:1 ratio of predator to prey. In each trial, eight *E. diaphana* individuals were evenly spaced in a circle around the edge of a large clear acrylic box (25 × 25 × 25 cm). The arena was placed on top of a white LED lightboard, which provided uniform illumination to facilitate visualization and analysis. Opaque black electrical tape was applied around the outside edges of the arena to block external visual stimuli. The animals were recorded from above using a Pro Stream Webcam 1080P HD at 1 FPS using Video Velocity software (Virginia City, NV, USA). The anemones were allowed to acclimate for 5 min and then eight *Berghia* were added to the center of the circle, equidistant from all anemones. After 20 min, the sizes of the groups and number of slugs that were not feeding were recorded and the slugs were returned to their home tanks. The maximum group size was the size of the largest group observed at any point during the experiment. The mean group size was calculated for the groups actively feeding at the end of the 20-min period (Table 1). The slugs used in these experiments were food-deprived for either 7 or 3 days depending on the experiment. Group sizes were counted by observers blind to the food-deprivation length. This experiment was repeated 15 times using new sets of eight anemone and 7-day food-deprived slug individuals each time, using a total of 120 different *Berghia* and *E. diaphana* individuals. This experiment was also repeated 13 times using 3-day food-deprived slugs for a total of 104 different *Berghia* and *E. diaphana* individuals.

**Table 1:** Details of each GF assay trial including the food-deprivation length of the slugs used, the maximum group size observed during the trial, the mean group size, and number of slugs not feeding 20 min after introduction to the arena

Trial	Food-deprivation length (days)	Number of slugs and anemones	Max group size	Mean group size	Number of slugs not feeding
1	7	8	2	1	7
2	7	8	4	2	2
3	7	8	4	2.5	3
4	7	8	1	1.4	1
5	7	8	4	1.75	1
6	7	8	3	1.6	0
7	7	8	6	3.5	1
8	7	8	1	1	6
9	7	8	3	2.33	1
10	7	8	3	1.75	1
11	7	8	3	2	2
12	7	8	3	1.33	3
13	7	8	3	1.75	1
14	7	8	3	1.75	1
15	7	8	2	1.67	3
16	3	8	4	2	0
17	3	8	3	2	2
18	3	8	5	2.33	1
19	3	8	2	1.5	2
20	3	8	3	1.67	3
21	3	8	6	4	0
22	3	8	3	2	0
23	3	8	3	2	0
24	3	8	2	1.33	0
25	3	8	2	1	4
26	3	8	2	1.4	1
27	3	8	2	1.5	2
28	3	8	2	1.33	0

### Two-alternative choice assays

To identify the cues *Berghia* might use to aggregate, we conducted a series of two-alternative choice assays. Each trial took place in a small square acrylic arena (7.62 cm × 7.62 cm × 2.54 cm) surrounded by opaque white window film to block external visual stimuli. The arena was illuminated from below by a white LED lightboard. Two anemones were placed in opposite corners of the arena and allowed to acclimate for 5 min before introducing a single *Berghia*. Slugs were acclimated in an identical arena using either plain ASW or anemone-treated water (ATW) depending on the experiment. ATW was prepared by incubating one anemone per 25 mL of ASW for at least 24 h, followed by filtration through a 0.22-µm hydrophilic polyethersulfone membrane (PES) filter (Millipore Sigma, Burlington, MA, USA). Acclimation in ATW for 5 min was used to prime the slugs and increase responsiveness to the food odor, increasing the likelihood of quickly selecting an anemone at the start of the trial.

During each trial, *Berghia* were placed equidistant from the two anemones using a placement guide to ensure consistent positioning. Trials were recorded from above, using the same equipment and frame rate as the GF assay. A trial ended when the slug contacted one of the anemones or after 30 min if no choice was made. After each trial, slugs were returned to their home tanks, and their choices were recorded. All slugs were food-deprived for 3 or 7 days, depending on the experiment.

The following experimental conditions were tested:

- *Slime trail (ST):* To test whether slugs followed an ST, a helper slug was allowed to navigate the arena until contacting one of two intact, size-matched anemones. The helper was removed after protruding its proboscis but before biting (Fig. 2A*i*).
- *Feeding conspecific (FC):* To test whether slugs were attracted to a conspecific actively feeding, a helper slug was allowed to feed on one of two anemones in a separate arena. Both anemones, along with the feeding helper, were then transferred to the testing arena. By transferring the anemone with the helper slug still attached, there was no ST leading to either anemone.
- *Feeding conspecific ± slime trail (FC ± ST):* To test combined cues, a helper slug navigated the arena and began feeding on one of two intact anemones. Both the feeding slug and its ST were present during the trial.
- *Bisected anemone (BA):* To determine whether slugs were attracted to injured anemones, slugs chose between an intact anemone and one bisected with a razor blade immediately before acclimation. All anemones were size matched, thus the BAs had a diameter that was half of the intact anemones also offered to each slug in this assay.
- *Munched anemone (MA):* We also tested a more naturalistic injury. To test attraction to an anemone fed on by a conspecific, a helper slug fed on one anemone for at least 10 min in a separate arena before the trial. These two anemones were then placed in opposite corners of the testing arena.

Helper slugs were free to choose between the same two anemones presented to the focal slugs, accounting for natural variation in anemone attractiveness across *Berghia* individuals. Trials were excluded if the focal slug failed to make a choice or, in cases involving an FC, if the helper slug stopped feeding before the focal slug made a selection. Anemones used in these experiments were size matched. The diameter of the chosen anemone and the other anemone were measured in ImageJ, and an ellipse was fit to the oral disc of each anemone using a single frame from the video where the anemone tentacles were outstretched. The area of the eclipse was used to estimate the diameter of each anemone. Trials where the focal slug did not make a choice within 30 min were omitted.

### Consistency of social preferences assay

To examine whether individual *Berghia* consistently preferred social or solitary feeding, we tested 32 slugs (4 groups of 8) after 7 days of food deprivation using the GF assay described earlier. Each slug was assigned an identifier, and its feeding behavior (group vs. solitary) was recorded. Slugs were then housed individually in clear plastic deli cups, provided with 24 h of ad libitum access to *E. diaphana*, followed by 7 days of food deprivation. Each slug was subsequently tested in the FC + ST two-alternative choice assay and its anemone choice was recorded. This cycle of feeding and 7 days of food deprivation was repeated until each slug completed four tests (see Fig. 4A). The total number of times each slug chose the social option (anemone with an FC and ST) was used to calculate a social preference score. Slugs unable to complete all four tests were excluded from analysis, thus only 24 individuals were analyzed.

### Statistical analysis

To statistically compare the group sizes observed with the null hypothesis that each slug chose independently of each other, we constructed a model with *m* slugs each selecting one of *n* anemones with equal probability (Equation 1). Using this model, we simulated a trial and calculated the mean and maximum group sizes. This was repeated for the same number of trials in each dataset and then the mean of the mean and maximum group sizes were calculated for each simulated dataset to create the null distribution. The experimental means of the mean and maximum group sizes were then compared to the null distribution. The probability of the null model producing the same result or larger than the experimental data was calculated for a *P* value, and 100,000 datasets were simulated for each statistical test.


(1) \begin{eqnarray*}
P\left( {\textit{slug}{\mathrm{\ }}\textit{selects}{\mathrm{\ }}\textit{anemone}{\mathrm{\ }}i} \right){\mathrm{\ }} = \frac{{{\mathrm{\ }}1}}{n}
\end{eqnarray*}

Additionally, we used the social dining model (SDM; often referred to as the “Chinese restaurant process”; Antoniak 1974; Pitman 2002) to estimate a concentration parameter representing the propensity of individuals to select an anemone with FCs. The SDM is a discrete process that simulates a set of individuals, *m*, each sequentially selecting a dining location, *i* (Equation 2). A concentration parameter, *α*, dictates how likely individuals are to select a dining location that is already occupied (Eq. 2). This model assumes that the number of anemones, *n*, is greater or equal to the number of slugs, *m*. The model also assumes that the order in which the slugs choose does not affect the final probability distribution. We estimated the concentration parameter using a bisection method to iteratively determine the parameter that fits the experimental data. We used this parameterized model to calculate a *P* value similarly to above.


(2) \begin{eqnarray*}
P\left( {\textit{slug}\ \textit{selects}\ \textit{anemone}\ i} \right) = \frac{{{m}_i}}{{m + \ \alpha }}
\end{eqnarray*}

In addition to the models described earlier, the GF assays for 3- and 7-day food-deprived animals were compared using a *t*-test. Before using *t*-tests, the assumptions for homogeneity of variances and normality were tested to confirm a parametric analysis was appropriate. For the two-alternative choice assays, the proportion of individuals that selected the manipulated anemone was compared to random chance (50%) using a binomial proportion test. A three-way analysis of variance (ANOVA) was used to compare the effect of the slugs’ choices, the acclimation method, and the anemone manipulation on the time it took them to make a choice. The latency to choose was log-transformed to normalize. To test whether slugs preferentially selected the larger anemones in any of the treatments, a nested ANOVA was used to assess whether the mean difference in the chosen anemone diameter from the anemone that was not chosen was different from zero.

The consistency of social preferences assay was analyzed using a Fisher's exact test. We also tested whether the distribution of social feeding scores (number of trials social option was selected) was bimodal using the Silverman (1981) critical bandwidth test as implemented by the *multimode* package (v1.5; Ameijeiras-Alonso et al. 2021). To assess the individual repeatability of two-alternative choice test outcomes, we estimated individual repeatability using the *rptR* package (v0.9.22; Stoffel et al. 2017) with their choice in the predator–prey ratio assay as a predictor and individual identity as a random intercept.

All modeling, visualization, and statistical analysis were performed in R version 4.2.3 (R Core Team 2023). Data manipulation used the *dplyr* (v1.1.4; Wickham et al. 2023) and *tidyr* (v1.3.0; Wickham et al. 2024a) packages. For visualization, we used the following packages: *ggplot2* (v3.4.4; Wickham 2024b), *ggpubr* (v0.6.0; Kassambara 2023a), *rstatix* (v0.7.2; Kassambara 2023b), and *cowplot* (v1.1.1; Wilke 2024). All code to reproduce this analysis and the figures in this paper are available on Github.

## Results

### *Berghia* fed in groups more than expected by random chance

**Fig. 1:**
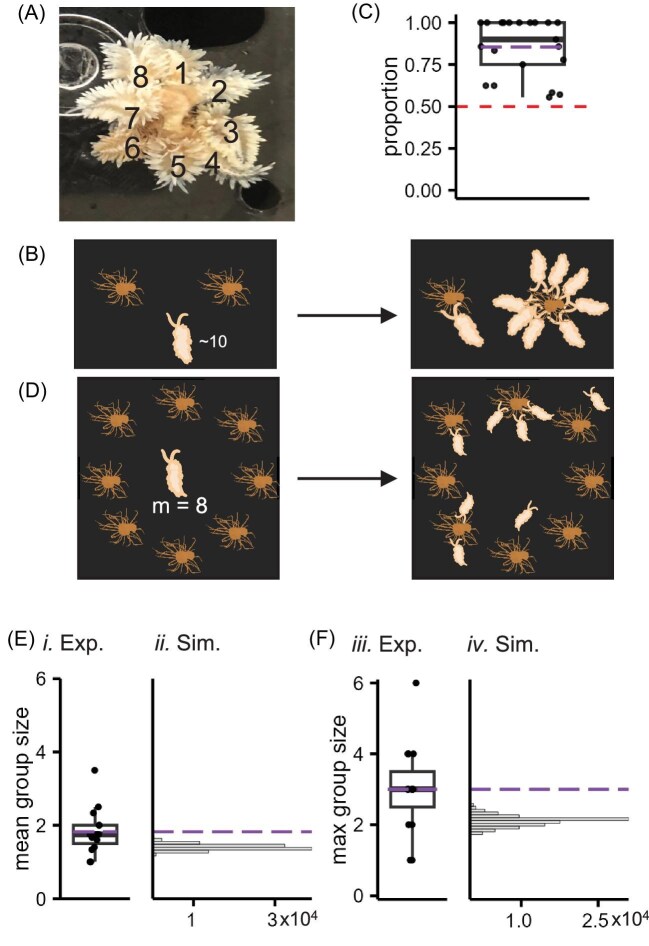
*Berghia stephanieae* form groups larger than if they each selected an anemone independently of each other. (**A**) Eight *Berghia* feeding on a single *E. diaphana* anemone in an aquarium. The slugs are numbered for clarity. (**B**) A schematic showing the experiment used to quantify the grouping during routine feeding in their home tanks. Two anemones were placed into each home tank, which contained about 10 slugs. After 20 min, the proportion of slugs feeding in the larger group was counted. (**C**) A boxplot of the proportion of slugs feeding on one of the two provided anemones. The slugs did not distribute evenly between the two anemones and tended to form large groups around one of them (*Z* = 231, *P* < 0.0001). Purple line represents the mean. Red dashed line represents even distribution between the anemones. (**D**) A schematic of the GF assay (GF). (**E**) A boxplot representing the mean group sizes observed in each trial (left) and a histogram of the mean group sizes for each simulated dataset with the same number of trials as the experimental data of the null hypothesis where each slug selects an anemone independently of each other (right). The purple dashed line represents the mean group size of the experimental dataset. The observed mean does not occur within the distribution of the simulated data. (**F**) The same plots as E, using the maximum group size observed. Similarly, the observed mean of the max group sizes in the dataset does not occur in the simulated data. The simulated datasets have units of 10,000 datasets.

This study was inspired by observations of large groups of slugs forming during feeding in the laboratory (Fig. 1A). This pattern was observed during every feeding and we quantified the distribution of slugs 20 min following a single routine feeding of 21 tanks of slugs, each containing about 8 slugs (median = 8, SD = ±1.7), and found that when fed with two anemones per tank, the slugs did not evenly distribute between the two anemones (Fig. 1B and C). A one-sample Wilcoxon signed-rank test indicated that the mean proportion of slugs feeding on one of the anemones, 0.85, was significantly different from 0.5 (*Z* = 231, *P* = 0.000052, effect size = 0.887). The anemones were randomly placed in the tanks and the slugs were not congregated in a single area of the tank; thus, this unequal grouping cannot be solely explained by each slug choosing the closest anemone. Often the entire tank of slugs would feed on a single anemone until it was gone and then move on to the other anemone.

To test whether *Berghia* feed on *E. diaphana* in groups even if they have the option to feed alone, we performed a GF assay. When given the opportunity to feed individually with a 1:1 ratio of *Berghia* to *E. diaphana* (Fig. 1D), *Berghia* fed in groups larger than expected if each individual *Berghia* was selecting an anemone independently of one another. Across 15 trials, the mean of the average group sizes observed in each trial was 1.82 (Fig. 1E*i*; median = 1.75, SD = ±0.62) and the mean of the maximum group sizes observed in each trial was 3 (Fig. 1E*iii*; median = 3, SD = ±1.25; Table 1).

To distinguish an active choice to aggregate around prey from random grouping, we simulated a scenario where each slug selected an anemone with equal probability (Equation 1), which is representative of conditions wherein each individual slug was selecting prey independently of one another, and 100,000 datasets with 15 trials each were simulated. There was no overlap between the experimental dataset mean and the simulation distribution; the experimental mean average group size was significantly more than expected by the simulated data (*P* = 0.00002; Fig. 1E*ii*) and the mean max group size of the experimental data was similarly larger than the simulated data (Fig. 1E*iv; P* = 0). Thus, the slugs are not choosing the anemones independently of each other.

### *Berghia* did not use the presence of feeding conspecifics or slime trails to select anemones to feed on

**Fig. 2:**
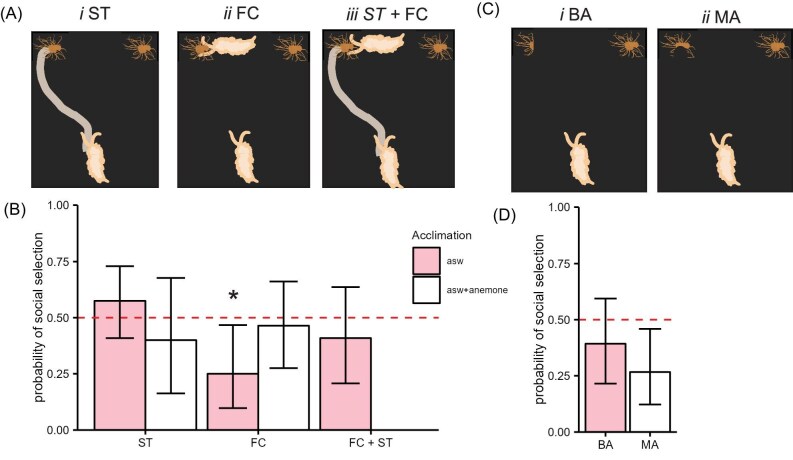
Behavior in two-alternative choice tasks. (**A**) Schematics of the choice between an intact anemone and an anemone with an ST, a feeding conspecific (FC) or both (FC + ST). (**B**) Bar plots showing the proportion of animals that selected the manipulated anemone for each of the choices depicted in A. The red dashed line indicates random choice. Error bars represent 95% credible intervals of the binomial test. Pink bars represent slugs that were acclimated in filtered ASW and white bars represent slugs acclimated in ATW. All choices were not significantly different from random chance, except FC when acclimated in ASW, which was selected lower than chance, meaning the slugs preferred an anemone without an FC (8/30, *P* = 0.016). (**C**) A schematic of the choice between an intact anemone and an anemone that had been cut in half (BA) and an anemone that had been previously fed on by a conspecific (MA). (**D**) Bar plots showing the same as in B. None was significantly different from random chance.

A series of two-alternative choice assays were performed to examine potential cues that *Berghia* could be using to aggregate (Table 2). One such cue is that the animals could be following the ST left by a conspecific. The slugs did not choose to feed on an anemone with an ST laid by a conspecific leading to it more than chance (*P* = 0.43, 23 out of 40). The slugs were also tested following an acclimation in ATW, to test whether prey scent would cause them to feed in groups due to heightened arousal. The target slug did not choose to feed on an anemone with the ST leading to it regardless of whether it was acclimated in ASW or in ATW (Fig. 2B; *P* = 0.61, 6 out of 15). When given the choice to feed on an anemone with an FC, the focal slug preferred the intact anemone (Fig. 2B; *P* = 0.023, 6 out of 24). However, this preference went away when the target slug was acclimated in ATW (Fig. 2B; *P* = 0.85, 13 out of 28).

**Table 2:** Details of the assays used in the two-alternative choice tests^a^

Assay	Food-deprivation length (days)	Acclimation	Sample size	Social selection	Binomial test *P* value	Number of omitted trials
ST	7 days	ASW	40	23	0.43	2
ST	7 days	ATW	15	6	0.61	1
ST	3 days	ATW	18	9	1.00	1
**FC**	**7 days**	**ASW**	**24**	**6**	**0.023**	**1**
FC	7 days	ATW	28	13	0.85	0
ST + FC	7 days	ASW	22	9	0.52	2
ST + FC	3 days	ATW	16	10	0.45	0
BA	7 days	ASW	28	11	0.35	2
**MA**	**7 days**	**ATW**	**30**	**8**	**0.016**	**1**
MA	3 days	ATW	17	7	0.63	1

^a^Abbreviations are consistent with those in the methods: ST = slime trail, FC = feeding conspecific, ST + FC = slime trail and feeding conspecific, BA = bisected anemone, MA = munched anemone. Acclimation used either artificial sea water (ASW) or anemone-treated artificial sea water (ATW).

To determine whether the slugs needed the combination of a slime trail plus a feeding conspecific (ST + FC), a helper slug was placed in the middle of the testing arena and allowed to navigate the arena until it began feeding on one of two intact, size-matched anemones. The target slug was then placed in the arena. There was no preference shown for either anemone despite the presence of both an ST and an FC (Fig. 2B; *P* = 0.52, 9 out of 22).

### *Berghia* did not prefer anemones that have been injured

The potential influence of kairomones from injured *E. diaphana* was tested with a two-alternative choice assay. *Berghia* showed no preference when given a choice between a BA and an intact anemone (Fig. 2D; *P* = 0.35, 11 out of 28). We tested whether slugs preferred an anemone that had been injured naturalistically by a conspecific (MA) and contrary to our prediction, slugs showed a preference for intact anemones over anemones that had been previously fed on by a conspecific (8/30, *P* = 0.016; Fig. 2D).

Although the slugs did not show a preference to various social cues, they might have contacted the manipulated anemone more quickly, which could lead to aggregation. Slugs that selected the social option did not do so in less time than animals that selected the control anemones for any of the treatments (Supplementary Fig. S1 and Supplementary Table S1). There was no effect of choice (*F*_(1)_ = 1.113, *P* = 0.293), nor a statistically significant interaction effect (*F*_(3,3)_ = 0.617, *P* = 0.605). Although it was an effect of the assay type on the latency to select an anemone (*F*_(3)_ = 8.851, *P* = 1.89e-05), this was not a main effect of interest (Supplementary Table S2). Slugs that were acclimated in ATW were faster to choose an anemone (*F*_(1)_ = 18.578, *P* = 2.88e-05), likely due to heightened arousal from the scent of their prey prior to entering the arena, which differs from previous findings that food-deprived slugs in an empty arena move slower when presented with a food odor (Quinlan and Katz 2023). This effect interacted significantly with their choice (*F*_(1,1)_ = 5.864, *P* = 0.0166), such that their latency was impacted the most when the slugs selected control anemone and ATW acclimation caused them to choose faster. There was no interaction effect between acclimation and manipulation (*F*_(1,3)_ = 0.734, *P* = 0.3931), nor was there a three-way interaction between the terms (*F*_(1,1,3)_ = 1.658, *P* = 0.1998). The mean difference in anemone diameter between the anemone choices was not significantly different from 0 for any of the manipulations (Supplementary Fig. S2; *F*_(6)_ = 0.594, *P* = 0.735).

### Social predation was not facilitated by intermediate levels of food deprivation

**Fig. 3:**
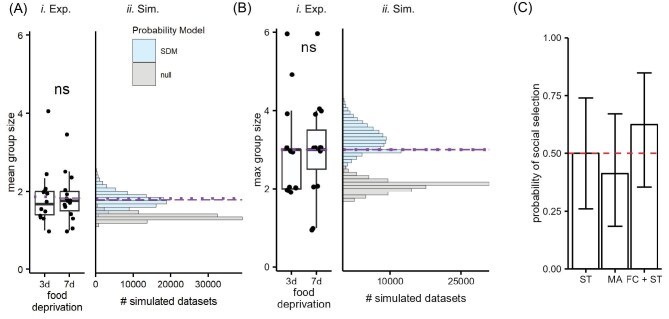
There is no difference in group size between intermediately food-deprived animals and 7-day food-deprived animals. (**A**) Boxplot of the mean group size for trials that were 3-day food-deprived and 7-day food-deprived (left). Histogram of the dataset mean of the mean group sizes observed in 10,000 simulated datasets (right). The light blue bars represent the parameterized SDM, and the gray bars represent the null model. The dotted purple line is the experimental dataset mean for the 3-day food-deprived animals and the dashed purple line is the experimental mean for the 7-day food-deprived animals. There is no difference between the experimental means and they fall within the SDM simulated dataset means and do not overlap with the null simulated dataset means. (**B**) Similar plots as A for the dataset mean of the maximum group sizes. (**C**) The probability of selecting the manipulated anemone in two-alternative choice assays comparing feeding conspecifics and slime trails (FC + ST), anemones previously fed on by a conspecific (MA), and anemones with ST. None was significantly different from random chance (0.5).

Animals might be changing their social feeding strategies because of a trade-off between food acquisition and injury. To test the prediction that social predation is more prevalent in animals that are intermediately hungry, we compared 3-day and 7-day food-deprived animals in a group-feeding assay. Across 13 trials, the mean of the average group sizes observed per trial was 1.85 for the 3-day group and 1.82 for the 7-day food-deprived group (Fig. 3A*i*; 3-day median = 1.67, SD = 0.75; 7-day median = 1.75, SD = 0.62). The mean maximum group size was 3.00 for both groups (Fig. 3B*i*; 3-day median = 3.00, SD = 1.25; 7-day median = 3.00, SD = 1.29; Table 1). The mean group sizes for the 3-days food-deprived animals were not significantly different from those of the 7-days food-deprived animals (Fig. 3A; *t* = 0.11074, *df* = 23.553, *P* = 0.9128). The maximum group sizes were also not significantly different (Fig. 3B; *t* = 0, *df* = 25.201, *P* = 1).

Similarly to the 7-day food-deprived data, we compared the 3-day food-deprived experimental data to the distribution of simulated dataset means of the mean and max group size when each slug chose an anemone independently of one another with equal probability (Equation 1). These simulated datasets had 13 trials each, like the experimental dataset (Fig. 3A*ii* and B*ii*). The observed mean average group size and the mean maximum group size were significantly larger than the simulations (mean *P* = 0.00037, max *P* = 0.00084). Thus, as predicted, 3-day food-deprived animals are also not choosing anemones independently of each other.

To test whether 3-day food-deprived animals socially feed more than 7-day food-deprived animals, we parameterized the SDM using the 3-day food-deprived dataset. The concentration parameter, α, was estimated to be 4.063. The distributions of the simulated dataset means for the mean and maximum group sizes included the experimental mean average group size (Fig. 3A*ii; P* = 1) and mean maximum group size (Fig. 3B*ii; P* = 1). Additionally, the 7-day food-deprived experimental mean of the mean and maximum group size was also within the simulated distribution parameterized with the 3-day food-deprived dataset. This indicates that the grouping as indicated by the α parameter is similar for both levels of food deprivation.

We also tested 3-day food-deprived animals in some of the two-alternative choice assays. Like the 7-day food-deprived animals, 3-day food-deprived slugs showed no preference for any of the cues (Fig. 3C). There were no differences between anemones with an ST (*P* = 1.00, 9 out of 18), anemones that were previously fed on by a conspecific (*P* = 0.63, 7 out of 17), or anemones with an FC and an ST (*P* = 0.45, 10 out of 16).

### *Berghia* did not show consistent individual preferences to feed in groups

**Fig. 4:**
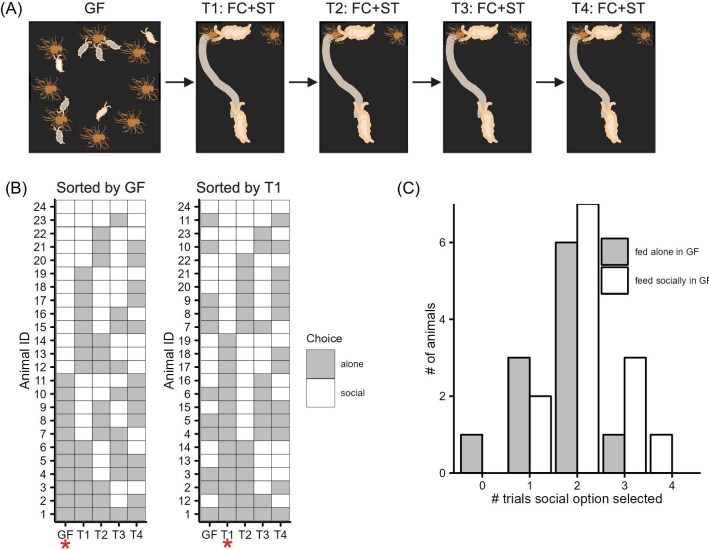
The choice to feed socially is not consistent within individuals. (**A**) Schematic showing the experimental design for this dataset. Animals were first tested in the GF assay and then individually labeled and housed. Then they were tested four times (T1–T4) in a two-alternative choice assay with a feeding conspecific and slime trail (FC + ST). (**B**) A plot showing the choices of each individual animal in the five different assays organized by their choice in the GF assay (left) and their choice in the T1 assay (right). (**C**) A histogram showing the number of animals that fed socially in the two-alternative assays (T1–T4) 0–4 times. White represents animals that fed alone in the GF assay and gray represents animals that fed socially in the GF assay. The distribution is not bimodal and animals seem to randomly switch between feeding socially and alone.

It is possible that the reason none of the cues tested were supported as a mechanism for social predation could be that individual slugs have consistent preferences to feed socially or not. This individual preference might be evenly distributed through the population; thus, a random sample from the population would appear to have no preference. Therefore, we gave individual identifiers to 32 slugs that were 7-day food-deprived and tested them in the group-feeding assay, recording whether each slug fed in a group or alone (GF; Fig. 4A). In this first test, 13 of 26 animals fed socially. Six animals were removed from the analysis because they did not complete all four of the subsequent tests.

These individual slugs were then tracked through four ST + FC trials each 7 days apart (Fig. 4A). Their choices in the subsequent assays were used to create a score for each animal that represented the total number of times each individual chose the social option (anemone with an FC and an ST). Their first choice was compared to subsequent choices. In the first two-alternative choice trial (T1), 10/26 of animals selected the social option and 8/13 of them had fed socially in the GF assay and 7/13 of them fed socially in the second two-alternative choice trial (T2; Fig. 4B). The choice to feed socially in the GF assay was not predictive of how many times an animal would select the social option in the two-alternative choice assays (Fisher's exact test, *P* = 0.1548). If individual animals had consistent preferences to feed in groups, we would also expect a bimodal distribution in the number of trials they selected the social option, but the distribution was unimodal (Silverman 1981 critical bandwidth test, critical bandwidth = 0.3612, *P* = 0.738; Fig. 4C). Their choices were not repeatable (R = 0, 95% confidence interval [CI] = 0., 0.136, *P* = 0.5).

## Discussion

Our study revealed that *Berghia* feed on their prey socially. This finding suggests that social predation in *Berghia* may serve an adaptive function. An alternative explanation is that the grouping behavior observed in these assays is not driven by social attraction but rather by differences in the attractiveness of the individual anemones. For example, in mosquitos some individual humans are more attractive due to their specific combinations of kairomones (Ellwanger et al. 2021; Giraldo et al. 2023). However, we can discount this hypothesis because if analogous kairomones exist in *E. diaphana*, some individual anemones would emit cues that universally increase their attractiveness to *Berghia*. If these combinations of cues reliably increased attraction across the species, the two-alternative choice assays that use helper slugs would have captured this effect and shown focal slugs selecting the same anemone as the helper slug more than random chance. Thus, differential prey attractiveness alone does not explain social predation in *Berghia*.

Contrary to our expectations, *Berghia* showed no preference for anemones associated with conspecific STs or active feeding by conspecifics in two-alternative choice assays. This result challenges the assumption that conspecific cues, such as mucus trails, drive aggregation at prey sites in this species. Nudibranchs, like other gastropods, rely on deposition of mucus that they glide on using cilia on their muscular foot. In terrestrial and aquatic gastropods, trail following is a mechanism that many species use to find mates (Ng et al. 2013), hunt other gastropods (Leonard and Lukowiak 1984; Patel et al. 2014), and otherwise aggregate (Bretz and Dimock 1983; Davies and Beckwith 1999). However, this was not a cue that mediated aggregation at anemones in two-alternative choice tests.

Conspecific cues are often key drivers of social behavior in other species, and may include the role of social influence, where the actions of conspecifics drive behavioral changes and/or shifts in motivational states of an individual (Whiten and Ham 1992; Webster and Fiorito 2001). For example, the presence of feeding crabs can stimulate feeding behavior in conspecifics (Kurta 1982). Similarly, meat traps for *Vespula germanica* wasps are facilitated by the presence of conspecifics at the trap (D'adamo et al. 2003). That said, the presence of an FC also was not sufficient to cause slugs to aggregate in this study.

The absence of attraction to these cues in *Berghia* could indicate that more complex or context-dependent signals facilitate group formation, such as a critical density of individuals or a threshold of sensory input not captured in our assays. The group feeding assay demonstrated that slugs often feed in pairs, and in cases where they feed in larger groups, the first slug to join a group must have responded to cues from a single conspecific. Thus, slugs must be able to respond to cues from a single other conspecific. This highlights the need for further exploration of alternative mechanisms, such as chemical signaling or tactile interactions, which may occur under natural conditions or at higher population densities.

Kairomones or alarm cues from prey can attract predators (Schoeppner and Relyea 2005; Aguilar-Marcelino et al. 2014). In other predatory-prey systems, kairomones released by injured prey have been shown to stimulate aggregation in nematode-hunting mites and frogs (Bilgrami 1994; South et al. 2020). However, in *Berghia* we found no evidence for attraction to injured anemones. Relationships between nudibranch predators and the alarm cues of their prey are not unheard of; the aeolid nudibranch *A. pappilosa* retains the alarm cue from the sea anemone *A. elegantissima* and uses this cue to evoke antipredator responses in other *A. elgantissima* individuals making them more susceptible to predation (Howe and Harris 1978). It is possible that injury-related chemical cues from anemones are less relevant to predation strategies in *Berghia* or that such cues are masked or altered in the controlled laboratory setting.

That said, the two-alternative choice assay may not be sufficient for identifying cues in social feeding because it captures only the initial attraction and choice. In the nematode *Caenorhabditis elegans*, injury induces social feeding through activation of nociceptive neurons (de Bono et al. 2002). Since the two-alternative choice assays were stopped at first contact between the slugs and their prey, it may not have allowed them to experience injury and then reevaluate their decision to ultimately select the other anemone. Individual *Berghia* may need to interact with their prey for a longer time period and then be allowed to make a selection. These results suggest that further experiments incorporating prolonged interactions and dynamic decision-making contexts could clarify whether injury or other post-contact cues play a role in the social predation of *Berghia*.

Food deprivation did not influence the propensity of *Berghia* to feed in groups, as slugs deprived for 3 or 7 days showed similar levels of social feeding. We hypothesized that comparing 7- and 3-day food-deprived animals would reveal a trade-off where hungrier animals were more likely to feed alone to maximize food intake at the risk of injury while intermediately hungry animals would feed in groups. In *Berghia*, the lack of a satiety effect suggests that social feeding is not primarily motivated by hunger but may instead serve other purposes, such as reducing predation risk or overcoming prey defenses. In this species, social feeding behaviors appear to be a strategy used by most individuals regardless of satiety.

These conflicting results led to the hypothesis that individual slugs have different likelihoods of using social predation as a strategy. If the animals preferring social predation and animals that prefer to feed alone are random in the overall population of *Berghia*, then randomly sampling from the population for the two-alternative choice assay would show a null result. However, we found no evidence of stable individual preferences for GF, unlike some other social predators across diverse taxa, which have stable individual roles across hunting bouts including social spiders and dolphins (Gazda et al. 2005; Dumke et al. 2016). Each individual is not foraging randomly. However, their roles seem to differ depending on context and specific predatory bout. This is similar to some fish species that exhibit social predation including false cleaner fish and the yellow saddle goat fish (Steinegger et al. 2018; Sato et al. 2024). The absence of such specialization in *Berghia* suggests that individuals may adopt temporary roles, such as scrounging or producing where individuals either use the strategy of joining groups feeding at specific food sources or locate their own sources, respectively (Vickery 2020). Through these experiments, we are able to fit *Berghia* into the social predation continua seen across animals and can perform further experiments to determine the ecological factors that promote these traits.

Social predation and grouping behaviors are likely influenced by population density and environmental conditions in the wild, neither of which have been extensively studied for this species. Future research should investigate natural populations in the field to better understand the ecological and evolutionary pressures shaping social predation in *Berghia*. Laboratory conditions, especially the uniform lighting used to facilitate analysis, are potential confounds of this experiment. The laboratory setting of this study may not fully capture the natural ecology of *Berghia*. It is possible that after generations of communal housing with limited food being introduced, this behavior was artificially selected for or learned, and would not be present in wild individuals. This should be further investigated, because if this behavior is specific to cultivated *Berghia*, it could provide an interesting basis for studying genetic or otherwise heritable changes in social behavior induced by environmental changes. In many social predators, cooperation enables individuals to subdue larger or more dangerous prey (Mukherjee and Heithaus 2013; MacNulty et al. 2014). It is possible that the social predation observed in *Berghia* allows them to overcome defenses and reduce injury from their radially symmetric prey by attacking from multiple sides. More studies are needed to determine whether injury is required to induce the use of aggregation cues and whether feeding socially reduces the injuries sustained by a single individual.

## Supplementary Material

obaf017_Supplemental_Files

## Data Availability

The datasets generated and analyzed in the current study are available on Github (https://github.com/OtterLabGroup/nudibranch_social_predation_2024). Behavioral videos are available upon request. A file containing statistical test results and additional plots is included. Additionally, there are three example videos of the group feeding assay included.
