# Supplementary material for: Social Predation by a Nudibranch Mollusc
Source: Integr Org Biol. 2025 Apr 28;7(1):obaf017. doi: 10.1093/iob/obaf017 (PMC12057695; doi:10.1093/iob/obaf017)
Supplement: obaf017_Supplemental_Files [file obaf017_supplemental_files.zip › supplemental 002 info.docx]

Electronic Supplementary Information

Social predation by a nudibranch mollusc

**Supplementary Table S1** ANOVA for latency to select an anemone during 2-choice alternative assays

|  | **Df** | **Sum squares** | **F-value** | **P-value** |
| --- | --- | --- | --- | --- |
| Choice | 1 | 0.165 | 0.165 | 0.293 |
| Assay Type | 3 | 3.930 | 8.751 | 1.89e-05 |
| Acclimation | 1 | 2.611 | 19.066 | 2.34e-05 |
| Choice: Assay Type | 3 | 0.274 | 0.617 | 0.605 |
| Choice: Acclimation | 1 | 0.803 | 5.864 | 0.0166 |
| Assay type: Acclimation | 1 | 0.100 | 0.734 | 0.3931 |
| Choice: Assay type: Acclimation | 1 | 0.227 | 0.346 | 0.1998 |

**Supplementary Table S2** Post-hoc comparisons of manipulations on latency to select an anemone during 2-alternative choice assays

| **Manipulation** | **Comparison** | **Mean Difference** | **SE** | **df** | **p-value** |
| --- | --- | --- | --- | --- | --- |
| BA | FC | -0.0696 | 0.0930 | 154 | 0.877 |
|  | MA | 0.352 | 0.112 | 154 | 0.0105 |
|  | ST | 0.113 | 0.091 | 154 | 0.604 |
| FC | MA | 0.422 | 0.100 | 154 | 0.0003 |
|  | ST | 0.1823 | 0.077 | 154 | 0.085 |
| MA | ST | -0.239 | 0.099 | 154 | 0.0758 |


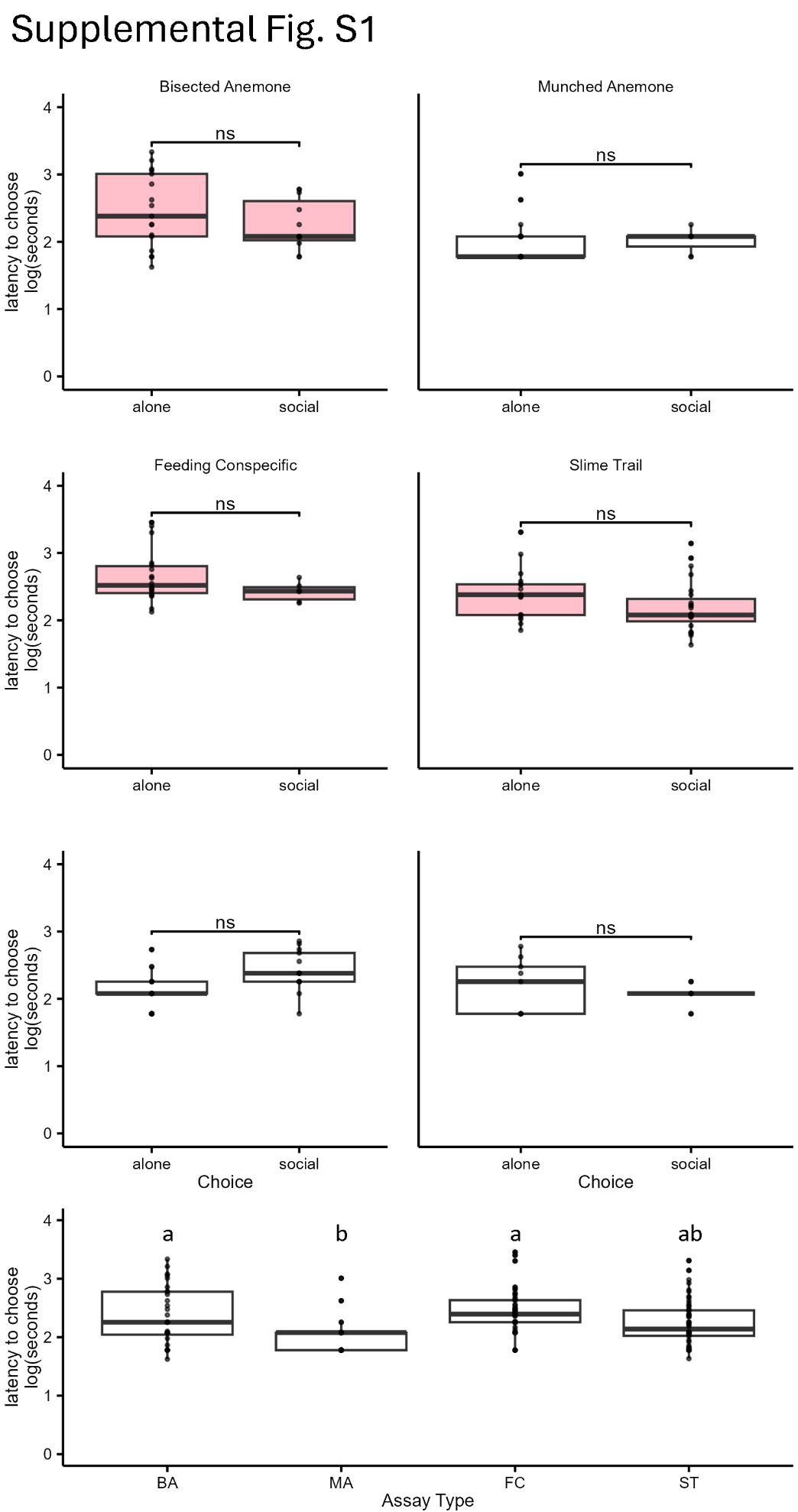


**Supplemental Figure S1** Animals that selected the social option in the 2-alternative choice assays did not choose faster than animals that did not select that option. The pink boxplots represent animals that were acclimated in ASW and the white boxplots represent animals that were acclimated in anemone-scented ASW. The data was log-transformed to normalize. The bottom boxplot shows the data with acclimation aggregated to show the results of pairwise post-hoc comparisons for the statistically significant predictor (assay type).

**
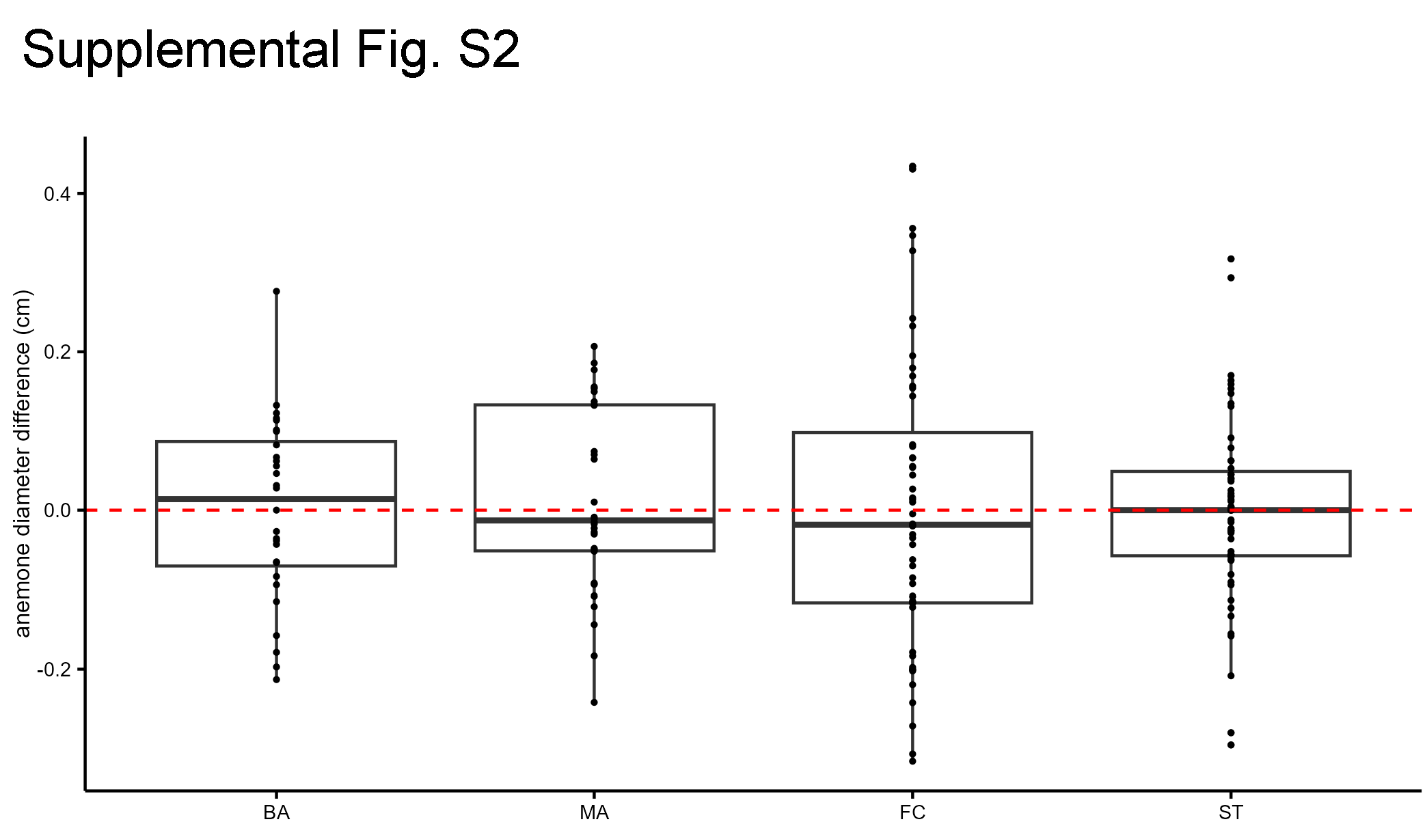
**

**Supplemental Figure S2** Animals did not consistently select larger or smaller anemones in the 2-alternative choice assays
